# Nitrate Reduction to Nitrite, Nitric Oxide and Ammonia by Gut Bacteria under Physiological Conditions

**DOI:** 10.1371/journal.pone.0119712

**Published:** 2015-03-24

**Authors:** Mauro Tiso, Alan N. Schechter

**Affiliations:** Molecular Medicine Branch, National Institute of Diabetes and Digestive and Kidney Diseases, National Institutes of Health, Bethesda, Maryland, United States of America; Albany Medical College, UNITED STATES

## Abstract

The biological nitrogen cycle involves step-wise reduction of nitrogen oxides to ammonium salts and oxidation of ammonia back to nitrites and nitrates by plants and bacteria. Neither process has been thought to have relevance to mammalian physiology; however in recent years the salivary bacterial reduction of nitrate to nitrite has been recognized as an important metabolic conversion in humans. Several enteric bacteria have also shown the ability of catalytic reduction of nitrate to ammonia *via* nitrite during dissimilatory respiration; however, the importance of this pathway in bacterial species colonizing the human intestine has been little studied. We measured nitrite, nitric oxide (NO) and ammonia formation in cultures of *Escherichia coli*, *Lactobacillus* and *Bifidobacterium* species grown at different sodium nitrate concentrations and oxygen levels. We found that the presence of 5 mM nitrate provided a growth benefit and induced both nitrite and ammonia generation in *E.coli* and *L.plantarum* bacteria grown at oxygen concentrations compatible with the content in the gastrointestinal tract. Nitrite and ammonia accumulated in the growth medium when at least 2.5 mM nitrate was present. Time-course curves suggest that nitrate is first converted to nitrite and subsequently to ammonia. Strains of *L.rhamnosus*, *L.acidophilus* and *B.longum infantis* grown with nitrate produced minor changes in nitrite or ammonia levels in the cultures. However, when supplied with exogenous nitrite, NO gas was readily produced independently of added nitrate. Bacterial production of lactic acid causes medium acidification that in turn generates NO by non-enzymatic nitrite reduction. In contrast, nitrite was converted to NO by *E.coli* cultures even at neutral pH. We suggest that the bacterial nitrate reduction to ammonia, as well as the related NO formation in the gut, could be an important aspect of the overall mammalian nitrate/nitrite/NO metabolism and is yet another way in which the microbiome links diet and health.

## Introduction

Nitric oxide (NO) is a highly diffusible short-lived free radical gas, permeating biomembranes with a wide range of physiological functions [1]. Nitrate (NO_3_^−^) and nitrite (NO_2_^−^) anions have long been identified as stable products of NO oxidation, but in recent years the nitrate—nitrite—nitric oxide reductive pathway has emerged as an alternative route to the classical enzymatic NO formation by oxidation of L-arginine with molecular oxygen [2,3]. Accumulating evidence indicates that oral commensal bacteria are responsible for the enzymatic reduction of inorganic nitrate to nitrite, which is then reduced to NO in the stomach under acidic conditions by non-enzymatic disproportionation [4], or in other tissues under physiological and hypoxic/ischemic conditions by several biochemical reactions catalyzed by a variety of enzymes and proteins [5,6]. Nitrate in the human intestine originates both from endogenous synthesis [7] and dietary products rich in nitrate [8]. Recent and past data have demonstrated that nitrate-rich diets increase plasma and tissue levels of nitrite [9,10], but this cannot be accounted solely by nitrate reduction from oral bacteria and other mechanisms have been implicated and are under investigation [11].

In healthy individuals dietary nitrate is usually well absorbed in the upper intestinal tract, however a considerable fraction of the daily nitrate intake (about 1/3) was found to reach the lower intestine while only 1% of it is recovered in the feces [12]. Research studies performed in the early 80’s by Tannenbaum and colleagues on the metabolic pathways of nitrate, both in humans and rats [13,14], showed that after diet supplementation with ^15^NO_3_^-^ about 50–60% of the ingested labeled nitrate was recovered in the urine while a small percentage (16% in rats and 3% in humans) appeared as ^15^NH_4_^+^ or [^15^N] urea and about 35% to 40% of the dose could not be recovered as excreted nitrogen-containing compounds. The metabolic fate of this unaccounted nitrate is still poorly understood. More recently, the normal bacterial flora has been shown to generate NO and gut luminal NO levels have been measured *in vivo* in rats [15,16]. Many enteric bacteria are also capable of catalytic reduction of nitrate to N_2_ gas (*denitrification*) under anaerobic conditions, or to ammonia *via* two-steps dissimilatory or assimilatory pathways [17,18]. We hypothesize that the nitrogen imbalance detected in the early metabolic studies cited above could be at least in part attributed to the gut microbiota conversion of nitrate to ammonia *via* nitrite reduction. The ammonia thus generated would likely be carried to the liver via the portal vein, where it can enter the urea cycle and be converted into urea and amino acids.

Very little information exists regarding O_2_ concentrations *in vivo* in the various fluids of the intestinal tract: typically, the O_2_ level at the luminal surface has been reported to range from 2% to 7% [19–21]. However, as oxygen diffuses from the tissues underlying the mucosa, microbial activity will reduce its content, and the lumen of the colon has been considered for many aspects an anaerobic region. In this study we investigated the formation of nitrite, NO and ammonia in cultures of representative species of gut bacteria grown with added nitrate under controlled oxygen concentrations existing in the human gastro-intestinal tract. In particular we selected *Escherichia coli*, the best understood enteric bacteria, and four different species of lactic acid bacteria (listed in **Table 1**) that have been previously shown to generate a substantial amount of NO when supplemented with 0.1 mM nitrite in anaerobic conditions (16). Our findings suggest that, in the presence of relative high physiological nitrate concentrations, *Escherichia coli* and *Lactobacillus plantarum*, two common bacterial species colonizing the human intestine, generate nitrite and subsequently ammonia in an oxygen-dependent fashion. The importance of this pathway *in vivo* demands further studies.

**Table 1:** Bacterial species and strains used in this study.

Species	Strain	pH^a^	Lactic acid (mM)^b^
*Escherichia coli*	MG1566	7.6	ND^c^
*Lactobacillus acidophilus*	ATCC 4356	4.2	46
*Lactobacillus plantarum*	ATCC BAA-793 (WCFS1)	5.0	19
*Lactobacillus rhamnosus*	ATCC 7469	4.8	25
*Bifidobacterium longum infantis*	ATCC 15697	3.9	51

Corresponding final pH of culture media and lactic acid concentration after 24 h growth at 2% O_2_ in LMRS are indicated.

^a^ pH of LMRS media after 24 h of bacterial growth. All are ± 0.1 (initial pH = 6.5)

^b^ Values are ± 1 mM and each sample was assayed in triplicates.

^c^ ND = non detectable

## Materials and Methods

### Growth Media and Reagents

The rich medium Luria-Bertani (LB) broth is usually the media of choice for fast growth of *E.coli*. However, we found that different batches of LB broth (from different vendors) contained a considerable, but variable, amount of ammonia and therefore it was not considered suitable for this study and used exclusively for the preparation of *E.coli* inoculum. Instead a modified LMRS broth (Lactobacilli de Man, Rogosa and Sharp broth), made without ammonium citrate (Anaerobe System, CA) was used despite *E.coli* growth rate being considerably lower (about 4–5 fold) than the rate in LB broth. Analyses of the LMRS medium for nitrite and ammonia indicated low concentrations present (respectively less than 1 μM and about 23 μM). Nitrate was added as a filter-sterilized solution. Lactic acid bacteria cultures were supplemented, when indicated, with hemin (stock solution: 0.5 mg/mL in 0.05 M NaOH) to a final concentration of 2.5 μg/mL and vitamin K_2_ (menaquinone-4) (stock solution: 2 mg/mL in ethanol) to a final concentration of 0.2 μg/mL. All reagents were purchased from Sigma-Aldrich unless otherwise specified.

### Organisms, Culture Conditions and Sample Preparation

A full list of bacteria strains used in this study is in **Table 1**. Stock culture collections were obtained from ATCC (Manassas, VA). Each strain was grown for individual experiments under the indicated oxygen concentration at 37°C. Bacterial cell concentration was monitored by measuring the optical density (OD) at 600 nm using a 1 cm pathlength cuvette. Typically, one hundred microliters of a 4 to 6 hours old inoculum of each strain with OD at 600 nm between 0.6–0.8 was added aseptically to 30–35 mL of broth in a 125 mL conical sterile flask, with shaking at 200 rpm. For high throughput, 6-well microtiter plates were filled with 4 mL medium/well, covered with breath-seals, shaken at 300 rpm and placed in a glovebox (Coy Laboratory Products, Grass Lake, MI). To vary the oxygen concentrations we supply the glovebox with different O_2_/N_2_ mixture adjusted accordingly and an oxygen sensor type Servoflex MiniMP-5200 was used to detect the exact oxygen concentration. At 0% O_2_, the glove box contained a gaseous atmosphere of about 2% H_2_ catalyst-deoxygenated nitrogen. The bacteria cultures were agitated using either a magnetic stirrer or a Micromixer Mxi4t. Samples were collected after 24 h or withdrawn at regular intervals, as indicated in the text, during time-course experiments and centrifuged at 10,000 × *g* for 15 min at 4°C. The cell-free supernatant and the cell pellet obtained were stored at −80°C. For further use the pellet was washed three times with 1 mL distilled water, weighted and re-suspended with enough PBS buffer to 10 mg/mL. The resulting suspension was sealed to prevent ammonia evaporation and used immediately to estimate ammonia and nitrite. The supernatant was used for nitrite and ammonia determination within 14 days. This prevented the loss of ammonia content in the samples as determined by comparison with standards prepared from 10 mM NH_4_Cl. To determine the colony forming units (CFU) one mL aliquot was collected and the 10^−2^, 10^−3^, 10^−4^ dilutions were plated onto LMRS agar (pH 6.5). All plates were incubated at 37°C until colonies were evident and counted manually.

### Analytical procedures

#### Determination of nitrite

To accurately measure nitrite concentration in cultures media and pellets after bacterial growth we used an acidic tri-iodide-based gas phase chemiluminescence method with a Sievers NO analyzer instrument (NOA, model 280i, GE Analytical Instruments, Boulder, CO, USA) as described previously [22].

#### Determination of ammonia

Ammonia concentrations in all culture samples were determined using two commercially available colorimetric assay kits optimized for 96 well plate reader (BioVision Inc., Milpitas, CA) respectively based on ammonia enzymatic conversion (OD at 570 nm) and a modified Berthelot non-enzymatic reaction (OD at 670 nm). Both are more reliable and sensitive that the method based on measuring NADPH oxidation (OD at 340 nm). Modified protocols were designed to limit interference due to low pH by buffering samples with 100 mM Tris-HCl at pH = 7.5 and diluting accordingly to assure the readings were within the standard curve range (prepared every time using NH_4_Cl standard solutions). For the non-enzymatic reaction, samples were deproteinized prior to testing using a 10kDa cutoff spin column filter.

#### Measurement of NO emission in the gas phase

NO gas liberated from live bacteria was measured in real-time by ozone-based chemiluminescence NO analyzer (CLD88Y; Eco Physics Inc., Ann Arbor, MI). Experiments were carried out as following: 10 to 100 mL of bacteria with their growth media at OD_600_ approximately 1.0 were placed in a spinner flask kept at 37°C while stirring and purged either with N_2_ gas for anaerobic conditions or with 2% O_2_ / 98% N_2_ gas mixture for low O_2_ conditions with the flow rate strictly regulated to 50 mL/min. For each experiment the colony-forming unit was obtained as described above and the amount of NO determined was expressed in ppb/10^9^ CFU. Once a stable baseline was established the indicated amount of nitrite was injected in the mixture as previously described [23]. To test for bacterial NOS activity nitrite injection was replaced with arginine or L-NAME as indicated. We verify that the release of NO into the gas phase from the solutions can be used as a continuous measurement for the NO production building a calibration curve with amounts of NO produced by the injection of sodium nitrite standards into an 0.1 M HCl acidified solution containing 10 mM ascorbic acid.

#### Determination of lactic acid

The total amount of D-/L-lactic acids produced during growth was determined enzymatically using a spectrophotometric (absorbance at 340 nm) commercial test kit (NZYTech, Lisbon, Portugal). The assay was performed on the supernatant of cultures obtained after centrifugation at 5000 rpm for 10 min and diluted appropriately.

### Statistical Analysis

Each experiment was performed in triplicate, and values are expressed as mean ± standard deviation (SD) from determinations representative of two or more independently grown bacterial cultures. Data were analyzed using Origin 8.1 (OriginLab Corp., Northampton, MA). To account for the small growth differences between each bacterial batch the values for nitrite and ammonia determined in the cell free supernatant were normalized using the OD at 600 nm measured after 24 h growth. Analysis for statistically significant differences among mean values was done, when applicable, using the one-way analysis of variance. Error bars represent the SD of the measurement.

## Results

### Effect of nitrate and oxygen on the growth patterns of *E.coli* in LMRS

**Fig 1:**
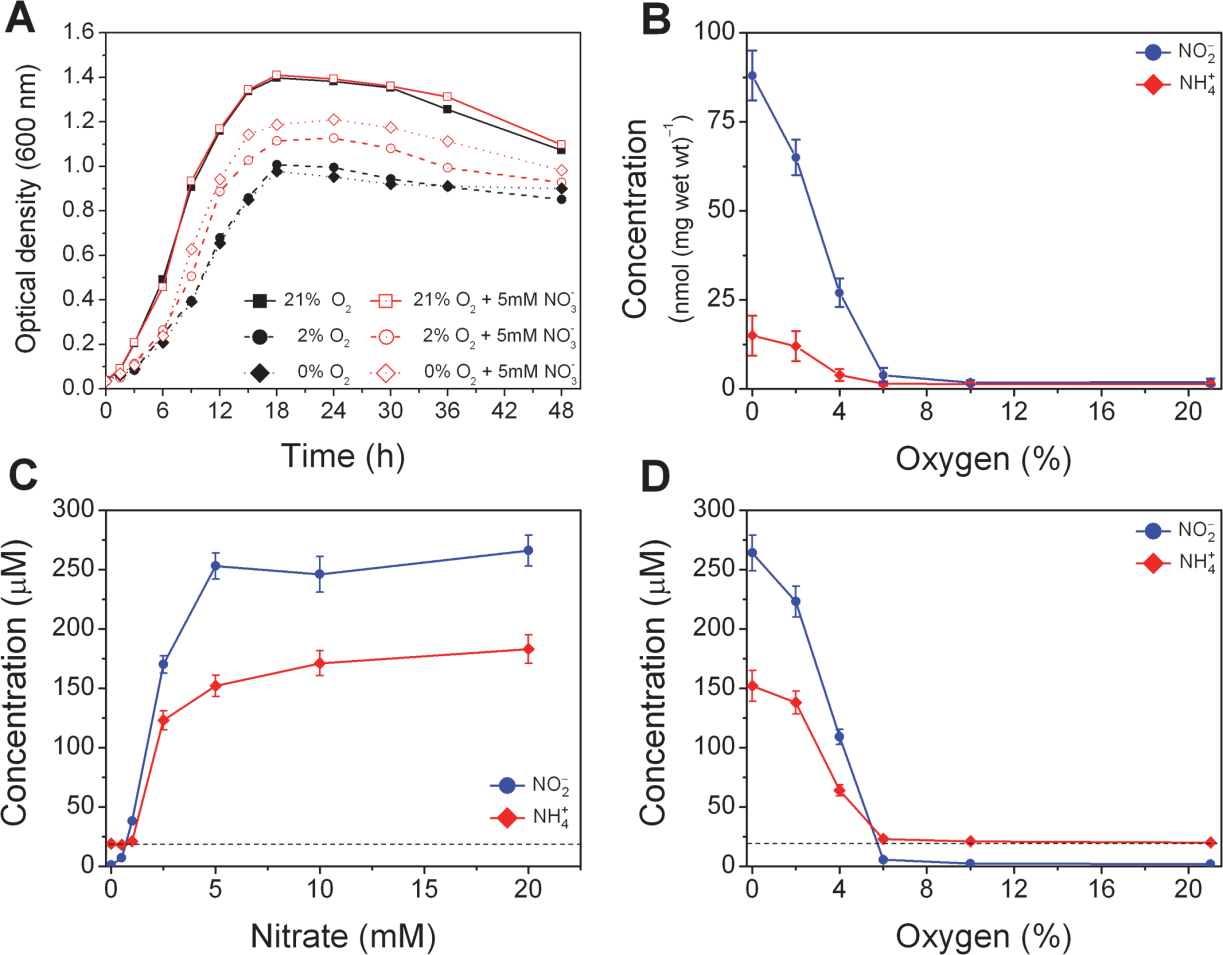
Nitrate and oxygen effect on *E.coli* bacterial cultures growth and formation of nitrite and ammonia. (A) Growth curves for *E.coli* MG1655 grown in the absence (black closed symbols) or in the presence of 5 mM nitrate (red open symbols) at 37°C in LMRS broth at 21%, 2%, and 0% O_2_ concentrations (respectively square, circle and diamond symbols). (B) Concentration of nitrite and ammonia (blue and red solid lines) in *E.coli* pellets after 24 h growth at different oxygen levels with 5 mM nitrate. (C) and (D) Respectively dependence on nitrate (at 0% O_2_) and oxygen (at 5 mM nitrate) of nitrite and ammonia concentrations in the cell-free culture media after 24h growth. The ammonia content of LMRS alone is indicated by the dashed lines. Values are means ± SD (n = 3). The average SD resulted smaller than the symbols dimensions (0.04 OD) and it is not shown for clarity.

We first compared *E.coli* MG1655 strain growth patterns in aerated, low oxygen (2%) and anaerobic cultures grown in modified LMRS broth supplemented with or without 5 mM nitrate, a concentration compatible with levels found in the upper intestinal tract of healthy volunteers and with values measured in the mouse intestinal mucus [24]. In **Fig. 1A** we plotted the OD of samples withdrawn at the same time intervals but different O_2_ levels: the aerobic condition (atmospheric concentration of 200 mbar or 21% O_2_) shows a shorter lag phase and, as expected, an earlier exponential growth phase starting at lower cell density than the 2% O_2_ and the anaerobic conditions (black lines with closed symbols), defined as concentrations of O_2_ below 5 mbar or 0.5% (about 0.1% in our experiments). The presence of 5 mM nitrate provided a clear growth benefit to *E.coli* cultures maintained at 2% O_2_ or in anaerobic conditions (red lines with open symbols) and partially restored the growth to levels found in the aerobic conditions.

### Effect of oxygen on *E.coli* production of nitrite and ammonia from nitrate

The *E.coli* genome encodes at least three distinct nitrate reductase enzymes [4] that are known to be expressed during anaerobic respiration [17]. These enzymes use nitrate as electron acceptor and produce nitrite which become toxic to the cell upon reaching high intracellular concentrations and is therefore transported outside the cell wall [25]. Alongside with transport *E.coli* expresses two nitrite reductase enzymes (Nrf and Nir) that detoxify NO_2_^-^ by rapidly converting it to ammonia through a six-electron reduction [18]. However, it is unknown how O_2_ levels affect these processes. We therefore measured both nitrite and ammonia in cell pellets (**Fig. 1B**) and cell–free supernatant (**Fig. 1D**) obtained from *E.coli* cultures grown for 24 h at 37°C and different oxygen concentrations with 5 mM nitrate added. In **Fig. 1B** the concentrations of nitrite in cell pellets (nmoles per mg of wet weight) show an increasing production starting at 4% O_2_ and reaching the maximum at 0% O_2_. The amount of ammonia detected followed the same trend but was at least 5 to 6 fold lower respect to the nitrite concentrations. The high nitrite/ammonia ratio (compared with values detected in the media) and the large errors in the ammonia measurements could be due to a considerable percentage of ammonia evaporating from the pellets while manipulating the samples at room temperature and for the remainder of this study we determined nitrate metabolites only in the cell-free supernatant. We then determined nitrite and ammonia in the media of *E.coli* cultures grown anaerobically for 24 h with added nitrate in the range 0 to 20 mM (**Fig. 1C**). Nitrite and ammonia concentrations remained steady when nitrate concentrations were lower or equal to 1.0 mM. However, when nitrate reached 2.5 mM both metabolites accumulated in the media and their concentrations were greatly increased and reached a maximum in the range 5–20 mM nitrate (to around 260 μM for NO_2_^−^ and 180 μM for NH_4_^+^).

### Nitrate reduction by lactic acid bacteria cultures

**Fig 2:**
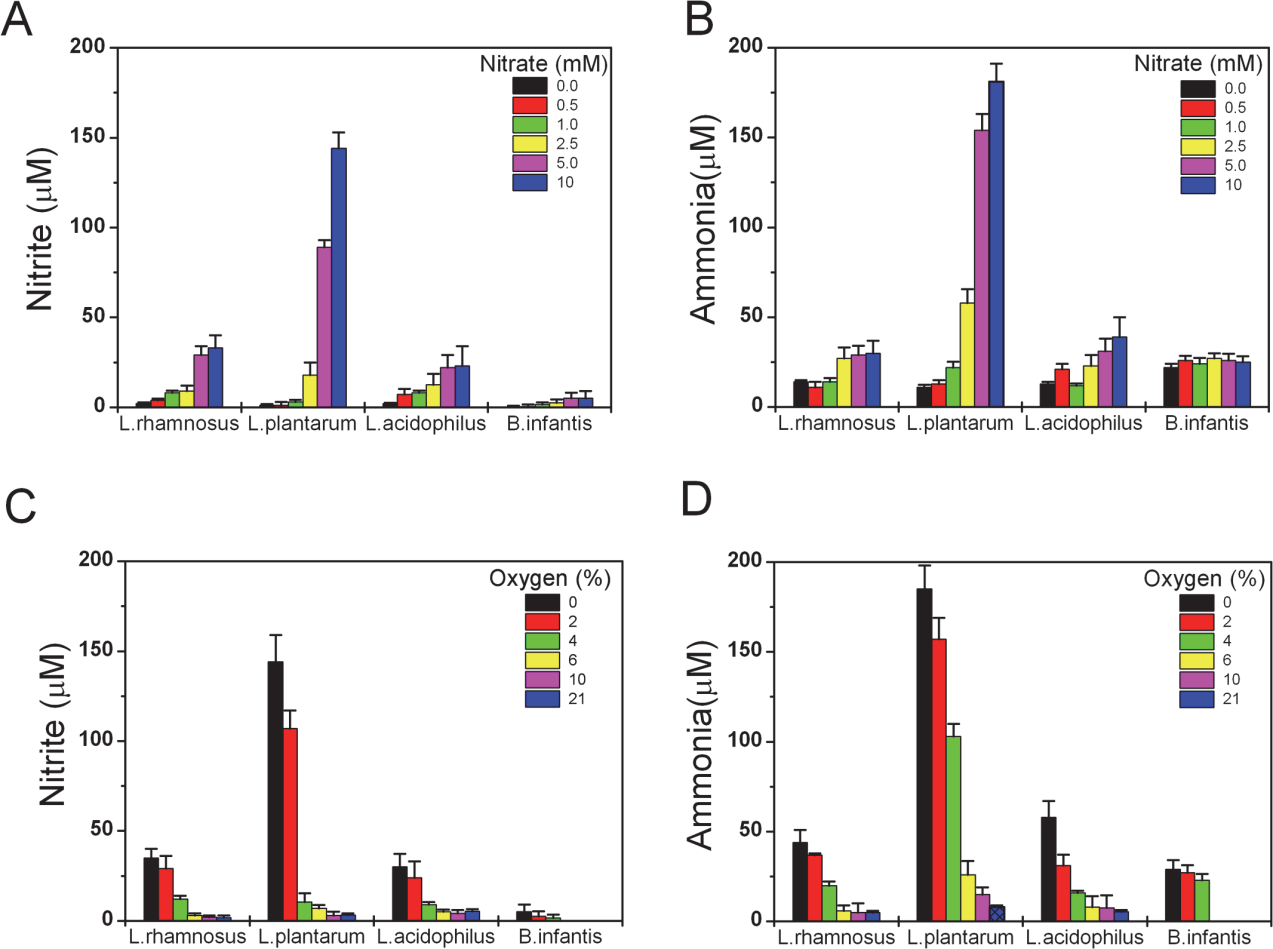
The effect of nitrate and oxygen gradients on the generation of nitrite and ammonia in different LAB cultures. Nitrite (A) and ammonia (B) concentrations were measured in LMRS media after 24 h growth at 2% O_2_ with supplementation of different nitrate concentrations (0 to 10 mM). Similarly in (C) and (D) nitrate was fixed at 5 mM and we measured nitrite and ammonia dependence on oxygen concentrations (0, 2, 4, 6, 10 and 21%). Each point represents the mean ± SD (n = 3).

Lactic acid bacteria (LAB) are facultative anaerobe organisms that grow in abundance in the digestive tract of vertebrate animals. LAB also represent some of the most commonly used probiotic bacteria and are extensively used for the production of fermented foods (yogurts, cheeses, sausages, pickles, etc.). It was believed that LAB depend strictly on a fermentative mode of metabolism since they do not possess heme containing enzymes essential for the respiratory chain. However, over the past 30 years it has been shown that many *Lactobacilli* species can incorporate heme from the environment and utilize menaquinones, also known as vitamins K_,_ to eventually perform respiration [26]. In this regards, it is important to note that *E.coli* synthesize both heme and vitamins K during growth facilitating membranous electron transfer. Brooijmans et al. [27] have also recently reported that exogenous addition of menaquinone 4 (vitamin K_2_) along with heme stimulate nitrate reduction in *L.plantarum*. We then grow single cultures of *L.rhamnosus*, *L.acidophilus*, *L.plantarum* and *B.longum* subsp. *infantis* species supplemented with heme and vitamin K_2_ and we compared the effect of oxygen and nitrate variations on the generation of nitrite and ammonia (**Fig. 2**). Although these strains are categorized as microaerophilic [28], in our experiments all strains grew well even at 21% O_2_ except for *B.longum* subsp. *infantis* which we confirmed as moderately aero-tolerant. First we compared nitrite and ammonia formation at different nitrate concentrations with the O_2_ level fixed at 2%, a partial pressure similar to the one found on the luminal surface of the intestinal mucosa (**Fig. 2 A** and **B**). Nitrate concentrations equal to or above 2.5 mM had a significant effect on nitrite and ammonia production only in the *L.plantarum* cultures. A smaller, but still considerable, effect on nitrite generation was observed in *L.rhamnosus* and *L.acidophilus* cultures and no significant changes were measured in *B.longum infantis*. We then fixed the nitrate concentration in the bacterial cultures to 5 mM, a level sufficient to show a clear effect on nitrite and ammonia generation both in *E.coli* and *L.plantarum*, and varied the O_2_ concentrations between 0% and 21% (the remaining being N_2_ gas) (**Fig. 2 C** and **D**). LAB cultures that grew at O_2_ levels equivalent or greater than 6% showed no significant changes in nitrite or ammonia. However when the O_2_ tension was 4% or lower nitrite and ammonia were both generated and excreted in the media and reached the highest concentrations in cultures grown under anaerobic conditions. Of note, cultures of *B*. *longum infantis* did not grow at, or above, 6% O_2_ and showed negligible content of nitrite and low ammonia independent of the oxygen level.

### Time-course of concurrent nitrite and ammonia formation

**Fig 3:**
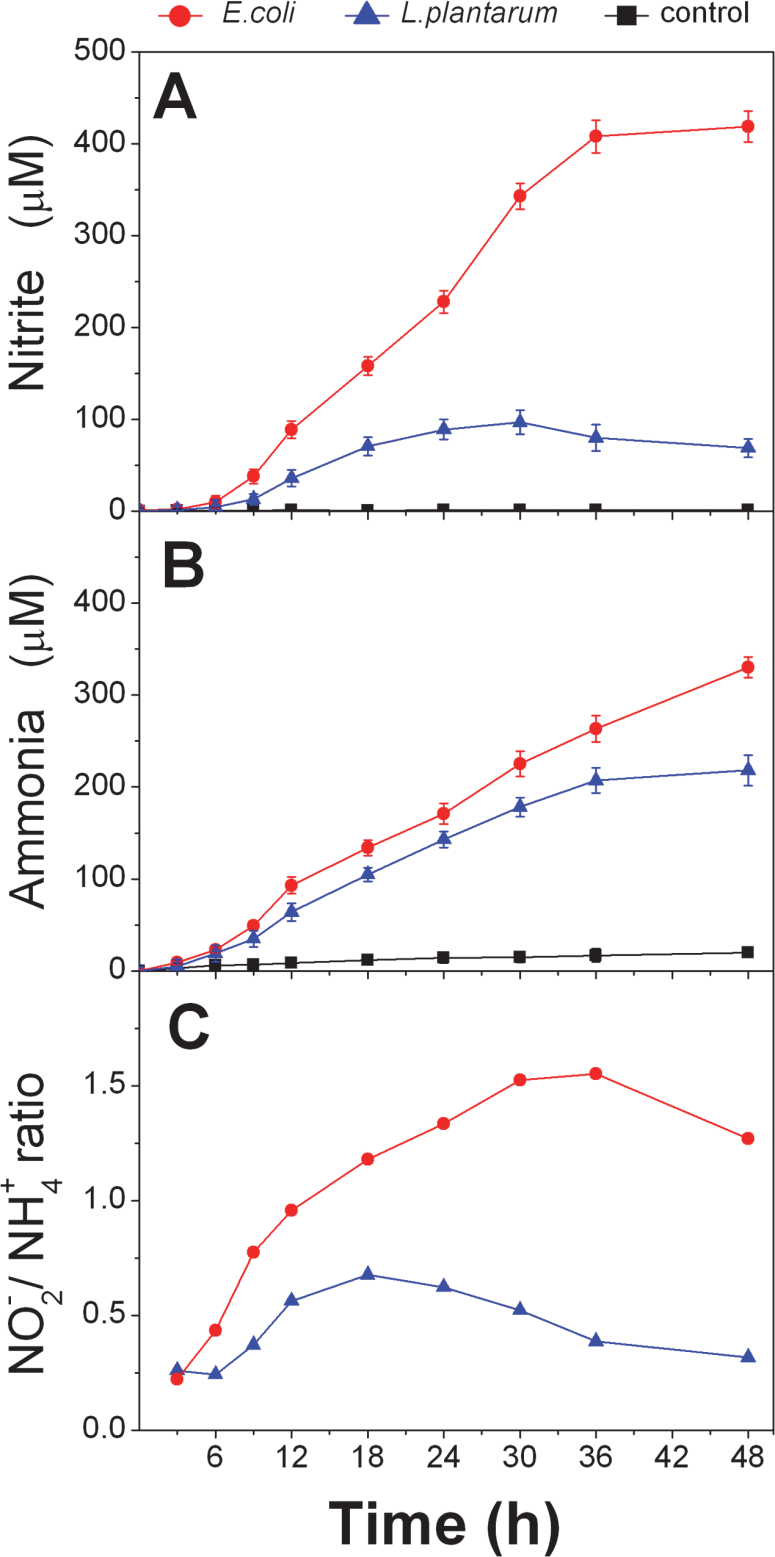
Time-course curves suggest that nitrate is first converted to nitrite and subsequently to ammonia. Samples from *E.coli* (red line with circles) and *L*. *plantarum* (blue line with triangles) cultures grown at 2% O_2_ with 5 mM nitrate were collected at regular intervals and analyzed for nitrite (panel A) and ammonia (panel B). (C). Ratio between nitrite and ammonia concentrations measured at each time point. Black lines represent *E.coli* cultures containing no added nitrate.

We measured relatively high concentrations of nitrite and ammonia in *E.coli* and *L.plantarum* cultures after 24 h growth in the presence of nitrate. Previous works on different *E*. *coli* strains have suggested that nitrate reductase molybdo-enzymes induced during anaerobic growth are responsible for nitrite (and possibly NO) formation and that the periplasmic nitrite reductase complex Nrf reduces nitrite directly to ammonium ion [29]. However this enzyme is subject to repression by oxygen and induction by high nitrite concentrations. To clarify the timeline of nitrite and ammonia formation under 2% O_2_, we determined the concentrations of these metabolites in LMRS supplemented with 5 mM nitrate during 48 h growth of *E.coli* and *L.plantarum* cultures, respectively indicated with red and blue solid lines **in Fig. 3**. In order to limit the effects of the different growth rates between species and batch cultures, in this experiment nitrate was added after the organisms exponential phase of growth upon cultures media reaching OD about 1.0 (time point = 0). We found that nitrite concentrations begun to increase within 3 to 6 hours after nitrate addition and accumulated steadily to reach a maximum in 30–36 h at about 0.4 mM for *E.coli* and 0.1 mM for *L.plantarum* (**Fig. 3A**). Similarly the ammonia concentrations plotted in **Fig. 3B** show well the almost linear and continuous increase measured between 9 and 30 h in both species but with different rates. To facilitate comparison between the 2 species the ammonia concentrations were normalized to zero at the time of nitrate addition (t = 0) and we reported also the values obtained for cultures without nitrate. Of particular note, nitrite concentration in *L.plantarum* increased until around 30 h and afterwards begun to decrease while ammonia kept increasing further. The formation of ammonia from nitrate indeed is proposed to occur via nitrite in two successive elementary steps, each with its rate law and characteristic kinetic parameters. However, nitrite can be reduced by other pathways (both chemical and enzymatic) and the correlation between the simultaneous multiple conversions would determine the nitrite/ammonia ratio. We plotted this ratio in **Fig. 3C** resulting approximately in asymmetric bell-shaped curves for both species. This result suggests that nitrate is first converted to nitrite and after some accumulation it is reduced to ammonia or other reduced nitrogen compounds.

### NO production by bacterial cell suspensions

**Fig 4:**
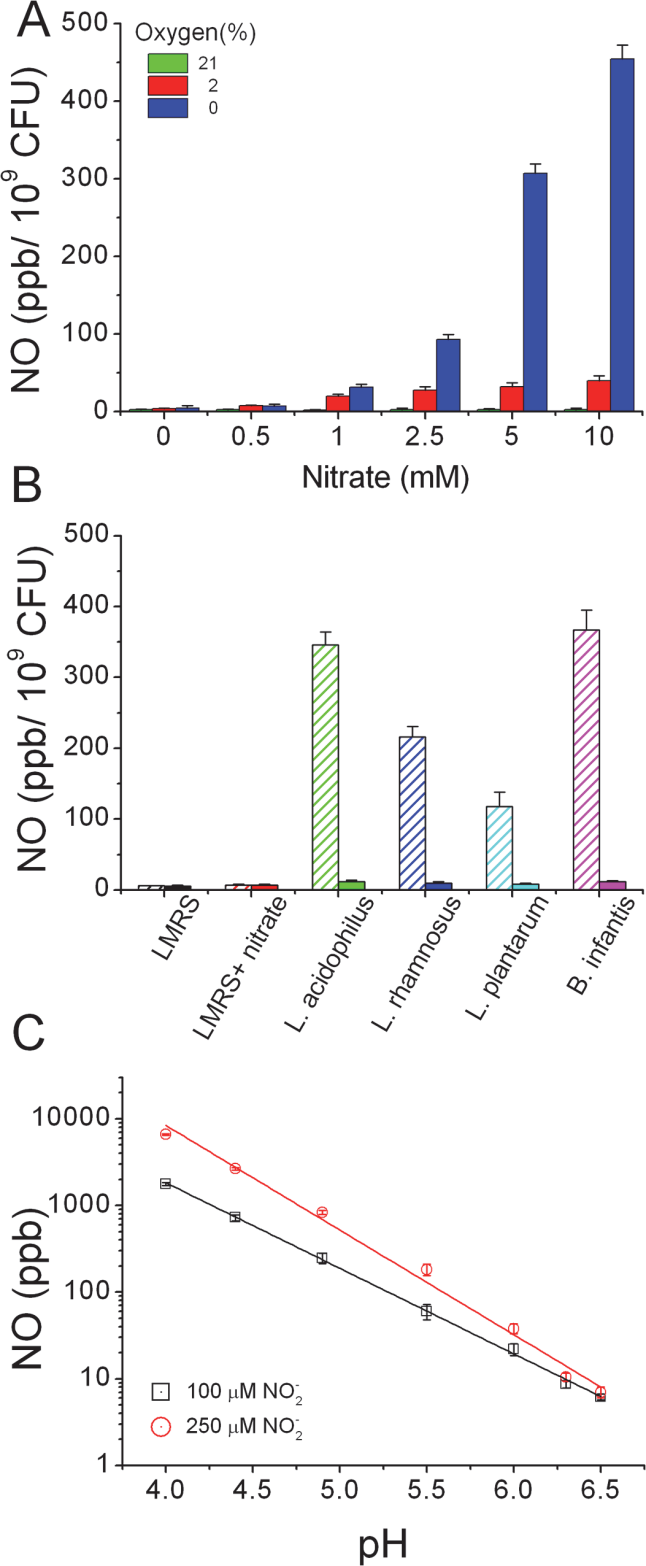
Bacterial NO generation and correlation with acidity of the growth medium. (A) Chemiluminescence detection of NO emission after injection of 100 μM nitrite in the vessel containing *E.coli* grown at different nitrate and oxygen conditions in modified LMRS broth for 24h. (B) Comparison of NO emission at 2% O_2_ as in panel A, but using LAB cultures. Diagonal patter and solid bars indicate respectively the values detected before and after bacteria were re-suspended in fresh media at pH = 6.5 (as described in text). (C) Quantification of the amount of NO detected after injection of 100 μM or 250 μM nitrite in fresh LMRS media at different pH obtained by acidification with concentrated L-lactic acid. All data represent mean ± SD in duplicates.

In 1988, Ji and Hollocher first observed the generation of NO from nitrite by E.coli in anaerobic conditions [30] and later concluded that nitrite-dependent NO production was due to the activity of the respiratory membrane-associated nitrate reductase enzymes [31]. However, several following studies on bacterial NO formation have proposed different mechanisms independent of respiratory denitrification such as arginine dependent bacterial NOS enzymatic activity, DNRA and non-enzymatic processes [15,32–34]. Here we used chemiluminescence techniques to measure NO in gas phase generated by a suspension of bacteria cells kept at 37°C under controlled O_2_ levels as described in “Materials and Methods”. In **Fig. 4A** we plotted the amount of NO detected after injection of 100 μM exogenous nitrite in a flask containing *E.coli* bacteria grown for 24 h in media supplemented with the indicated nitrate concentration. Under 2% O_2_ small amounts of NO (< 40 ppb/10^9^ CFU) were detected almost independently from the concentration of nitrate supplied. Inversely, much larger quantities were measured in analogous experiments after anaerobic growth and the response roughly correlated with the increasing nitrate concentrations. These results indicate that at least 2 processes producing NO are present in *E.coli*: one predominates at 2% O_2_ tension and the other in anaerobic atmosphere.

In **Fig. 4B** we then compared the generation of NO from the LAB species considered in this study and grown as described earlier with 5 mM nitrate at 2% O_2_. All the cultures produced a considerable but variable amount of NO after 100 μM nitrite injection, while the same addition to fresh LMRS, with or without nitrate, had no effect (bars with oblique lines). LAB cultures are well known to produce substantial acidification of the media due to the fermentation of glucose primarily to lactic acid and we confirmed its formation in large amounts (mM) by direct detection. The concentrations measured at 24 h are reported in **Table 1** together with the corresponding culture broth final pH. The initial pH (6.5) decreased variably to values between 3.9 and 5.0 depending on the LAB species and the difference was the least for *L*. *plantarum*. To elucidate if this acidification could be responsible for the generation of NO by the known non-enzymatic nitrite disproportionation, each bacterial preparation was split in equal volumes (10 ml) and tested before and after the replacement of its growth media with fresh LMRS by short centrifugation and decantation. This procedure resulted in the almost complete halt of NO generation in all LAB (solid bars in **Fig. 4B**) but only partially in *E.coli* cultures. Lysis by brief sonication in fresh media of the LAB cells to extract the cytosolic enzymes did not restored the generation of NO after injection of nitrite. We concluded that LAB production of lactic acid causes sufficient medium acidification to induce the chemical nitrite conversion to NO, instead this process in *E.coli* is enzymatic and occurs around pH 6.5. To verify our conclusions we measured the NO generation from 100 μM and 250 μM nitrite added to fresh LMRS medium containing sufficient L-lactic acid to adjust its final pH in the range 4.0–6.3 (**Fig. 4C**). Increasing amounts of NO were produced by the non-enzymatic nitrite disproportionation as the media pH decreased. The logarithmic plot of the total amount of NO detected (ppb) versus the pH revealed a linear correlation, similar to previously reported results for acidified nitrite solution in MRS broth or phosphate buffer [32]. Finally, we examined if NO was generated by bacterial NOS supplementing the cultures above with either 100 μM or 500 μM L-arginine, however this produced no appreciable changes in NO production independently from the oxygen and nitrate concentrations used (data not shown). Furthermore, addition of the NOS inhibitor L-NAME (100 μM) did not produce any decrease in the NO signal, which is expected in the presence of NOS enzymatic conversion (data not shown).

## Discussion

The human microbiota comprises more than a thousand distinct bacterial species [35] and plays a major role in human health by promoting nutrient supply, preventing pathogen colonization and shaping and maintaining normal mucosal immunity. Commensal gut bacteria have recently been appreciated as having a true symbiotic relationship with the host [36,37]; within this large pool of bacteria, probiotic supplements containing LAB (i.e. *Lactobacilli* and *Bifidobacteria*) have been claimed to have a variety of beneficial effects on human health, such as prevention of diarrhea and inflammatory bowel disease or prophylaxis of urogenital infections [38]. However, our knowledge of the biochemical roles that specific species and strains play in human health and disease is severely limited. In this study we aimed to advance the understanding of the nitrate reduction pathways in selected common bacterial species colonizing the human intestine using *in vitro* conditions compatible with nitrate-rich diets and oxygen levels found on the mucosal surfaces of the GI tract. The primary findings of our investigation indicate that: 1) *E.coli*, a facultative anaerobe, convert nitrate to nitrite and subsequently to ammonia which progressively accumulates in culture media; 2) *L.plantarum*, a fermentative bacteria, grown in the presence of exogenous heme and vitamin K_2_ perform similar processes; 3) *E.coli* enzymes generate significant NO from nitrite only under anaerobic conditions; 4) All LAB cultures examined generate large amounts of lactic acid causing sufficient acidification of culture media to drive nitrite disproportionation to NO.

Most eukaryotes derive their energy primarily through oxidative phosphorylation and must breathe O_2_ for the formation of ATP, however many enteric bacteria, including *E.coli* K12 strains, can use NO_3_^-^ as an alternative electron acceptor when O_2_ is limiting and nitrate is plentiful [39]. *E.coli* represents the model member of the Enterobacteriaceae and although this family constitutes only a small fraction of the gut microbiota, it is particular important because certain strains can cause illnesses. It has also been recently shown that nitrate generated as a by-product of host inflammation can be used by *E.coli* during respiration to confer a growth benefit and out-compete microbes residing in the colon that rely only on fermentation [40]. *L*. *plantarum* is considered a safe probiotic and is commonly found in the mammalian intestinal tract as well as in the human saliva where nitrates are known to accumulate to millimolar levels due to the entero-salivary cycle of nitrate, which accounts for about 25% of the overall circulating nitrate [11]. This bacterium presents the typical facultative heterofermentative pathway of the LAB family but, unique to this species, genes that encode a putative nitrate-reductase system (narGHJI) were recently identified in the *L*. *plantarum* WCFS1 genome, which suggests that it is capable of using nitrate as an electron acceptor [27]. Indeed a recently published genetic analysis of *L.plantarum* has highlighted its enormous diverse and versatile metabolic capability [41].

In our experiments significant nitrate reductase activity was detected both in *E.coli*, and *L*. *plantarum* as the oxygen tension decreases from atmospheric level towards zero. On the contrary, *B.longum infantis*, a micro-aero tolerant anaerobe of infant gastrointestinal tract origin, showed no ability to reduce nitrate even at high concentrations. *Bifidobacteria* represent up to 90% of the bacteria of an infant’s GI tract and our results are in accordance with the observation that human breast milk, which presents particularly high levels of nitrite [42], provides a dietary source for nitrite prior to the establishment of lingual and gut microbiota capable of nitrate reduction that are normally found in the adult flora.

In Fig. 1 we showed that *E.coli* cultures containing 5 mM NO_3_^−^ had a competitive growth advantage with respect to cultures with no nitrate added and then we determined the effect of oxygen and nitrate gradients on the production of nitrite and ammonia. Our results indicate that approximately 2.5 mM NO_3_^−^ at either 4% or lower O_2_ is sufficient to induce the expression of nitrate reductase enzymes and that after 24 h a considerable amount of nitrite accumulated both inside *E.coli* cells as well as in the culture media. A detailed molecular analysis of the regulation of the bacterial enzymatic activities transcends the scope of this study, however it is well known that *E.coli* K12 strains express three molybdenum-containing nitrate reductases [17] and that tungsten can deactivate these enzymes by replacing the molybdenum atom at the active site [43]. We found that the addition of 300 μM tungsten oxide to cultures grown as in experiments reported in Fig. 1 almost completely abolished the formation of nitrite (data not shown). Thus, we believe that molybdenum dependent nitrate reductases are responsible for the crucial step in the formation of nitrite. It is also important to note that *E.coli*, as well as many other species of bacteria, are susceptible to nitrite toxicity due to the formation of metal-nitrosyl complexes and they minimize this toxicity by the coordinate induction of a nitrite membrane transporter [25] and other enzymes that mediate nitrite reduction. A complete description of the *E.coli* nitrate and nitrite reductase enzymes genes and operons regulation and expression can be find in excellent publications by Stewart [29] and Cole [44].

### Nitrogen oxides reduction pathways in the human gut

**Fig 5:**
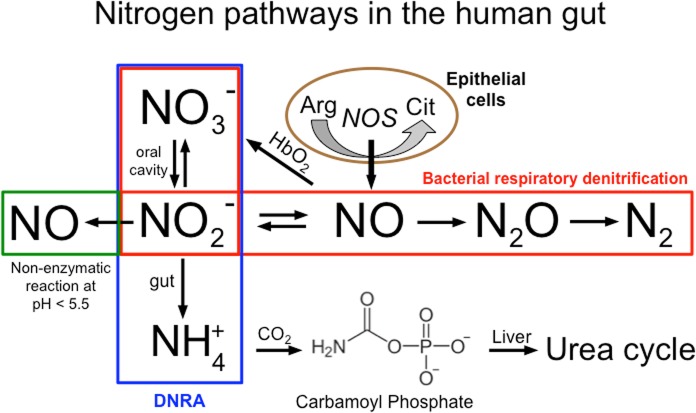
Schematic representation of the link between different pathways of nitrogen oxides reduction in the human gut and the fate of ammonia. Each colored box represents a distinct pathway: Bacterial respiratory denitrification to dinitrogen in red box: the dissimilatory nitrate reduction to ammonia (DNRA) in blue box and the non-enzymatic conversion of nitrite to NO in green box (this route become significant only at pH<5.5). The endogenous L-arginine/NO synthase pathway from epithelial cells of the intestinal mucosa lining is also noted.

The presence of nitrate and nitrite in the lower GI tract depends on numerous aspects including the types of bacteria colonizing the gut and the intricate balance between diet and the nitrogen oxides metabolic pathways. However the endogenous production of nitrate from NO oxidation (mainly via reaction with oxy-hemoglobin) has long been recognized to be an order of magnitude greater than dietary intake as shown in the late 1970s [7] and more recently in studies using eNOS-deficient mice [45]. In the schematic representation of **Fig. 5** we have summarized the link between bacterial respiratory denitrification, nitrogen oxides reduction to ammonia, the endogenous L-arginine/NO synthase pathway and the non-enzymatic nitrite reduction to NO. In the denitrification process (red box), nitrate is reduced to nitrogen gas (N_2_) in a four steps process in which nitrite, NO and nitrous oxide are electron acceptors in energy generating reactions. Recently a complete denitrification pathway, leading to production of N_2_, has been proved to exist in human dental plaque [46] and while it is still consider to be of minor importance in humans, we speculate that it might have an important role under very low oxygen tension in the presence of nitrate and the formation of N_2_ cannot be excluded in the human gut. Denitrification and dissimilatory nitrate reduction to ammonia (DNRA, blue box) share the first nitrate to nitrite reduction step and several classes of nitrate reductases have been associated with this reaction. In DNRA the second step is the direct nitrite to ammonia six electrons reduction, which does not provide energy but is a fairly common detoxification process in facultative anaerobic bacteria. DNRA has been suggested to represent the major route of nitrate metabolism in the rumen of mammals [47]. This study identified ammonia as a product of nitrate reduction in *E.coli* and *Lactobacilli* bacteria grown in the presence of mM nitrate at 4% oxygen or lower levels.

Respiratory denitrification can also generate small but relevant amounts of NO as an intermediate product and has been implicated in the bacterial NO production in the gut [32,33]. Other possible routes yielding NO are the acidic conversion of nitrite (green box) and the oxidation of L-arginine by NOS enzymes (brown circle). In Fig. 4C we examined the proton dependency of the non-enzymatic disproportionation of nitrite and showed that it becomes relevant only when the intracellular or body fluids pH is lower than 5.5. Our results also excluded the presence of active NOS enzymes in *E.coli* and *L.plantarum* however intestinal epithelial cells are known to produce NO through expression of both the endothelial and inducible NOS isoforms. Interestingly, NO production in the gut could be triggered also by the enzymatic processes of peroxidases, which are abundant on the cells of the gut mucosa and have been shown to use nitrite as a substrate to produce NO as part of their antibacterial action.

We believe that all these different nitrate reduction pathways may coexist and occur simultaneously however it is likely that only one metabolite would predominate depending on the specific physiological conditions.

### Physiological significance of NO formation by bacterial nitrate reduction

Dietary nitrate and nitrite are still pictured as possible toxic substances in many studies despite the mounting evidence that NO production from these ions has important beneficial implications for cardiovascular, immune and gastrointestinal functions [8,48,49]. In the gut NO serves several physiological functions such as regulation of mucosal blood flow, intestinal mobility and mucus thickness. The chronic overproduction of NO has also been associated with inflammatory bowel disease and is likely to inhibit growth of a wide variety of bacterial species. Previous studies left unclear how gut bacteria produced NO, however Sobko et al. [15] showed that in contrast to conventional rats, NO levels in the intestine of germ-free rats are extremely low and when inoculated with normal bacteria flora the observed NO production increased 10 fold.

In our experiments oxygen and proton concentrations determined the specific route of nitrate reduction to NO. The results presented in Fig. 4A indicate that *E.coli* is capable of enzymatic NO activity under anaerobic conditions with nitrate concentrations greater than 1 mM possibly *via* denitrification or the periplasmic cytochrome c nitrite reductase enzyme (Nrf) as proposed by Corker and Poole [33]. This NO generation, however is greatly reduced at 2% oxygen and become nitrate independent. Importantly, our data are consistent with the report by Sobko and colleagues that *E*. *coli* generated insignificant NO levels during 24 h incubation with 0.1 mM nitrate [15]. LAB produced considerable amounts of NO in response to the acidification of the media due to the accumulation of lactic acid. Replacing the growth media with fresh LMRS (pH = 6.5) almost completely blocked the LAB cultures ability to convert nitrite to NO but not in *E.coli*. Measurements of the intestine pH ranges between 5.7 and 7.5, thus *in vivo* nitrite disproportionation is probably a minor and localized aspect of the NO production. Inversely, this path is a well-established phenomenon in the acidic environment of the stomach (pH about 3).

In summary we suggest that the NO generated by gut bacteria in proximity of the intestinal mucosa may either exert the beneficial effects noted above or at higher levels, interfere with these functions. Thus bacterial NO formation in the gut can be regarded as modulator of both physiological and pathological effects.

### Physiological implications of bacterial ammonia formation for health

Colonic bacteria have been known to produce ammonia from amino acid deamination or via urease, the hydrolysis of urea into carbon dioxide and ammonia since the seminal studies of Vince et al. in the early 1970s [50]. More recently Cole and colleagues reported that the major product of nitrite reduction in *E.coli* is ammonia with about only 1% being reduced to NO at neutral pH [51]. The results obtained in our study suggest that at least certain common intestinal bacteria primarily reduce nitrite to ammonia rather than NO. In healthy subjects, under ordinary physiological conditions, the bulk of ammonia generated in the lower GI tract is then excreted in the body fluids and metabolized by the liver hepatocytes where ammonia and carbon dioxide are enzymatically converted to carbamoyl phosphate, which enters the series of reactions called the “Urea Cycle” leading to urea formation and its elimination by the kidney (see **Fig. 5**). The normal plasma concentration of ammonia is in the range of 10–35 μM, however, when ammonia production is excessive, portal blood-carrying ammonia can bypass the liver leading to hyperammonemia [52,53]. Ammonia in the blood freely permeates through the blood-brain barrier and high levels (>100 μM) have toxic effects on the central nervous system leading to encephalopathy and eventually coma. Patients with liver cirrhosis very frequently develop hepatic encephalopathy (HE) [54]. In the absence of liver failure, hyperammonemic coma has been attributed to sepsis by urease capable microorganisms such as *Klebsiella pneumonia* [55]. Classic therapeutic approaches for HE involve the reduction of systemic ammonia levels via antibiotic treatment (to kill intestinal ammonia producing bacteria) and administration of non-absorbable sugars, such as lactulose and lactitol [56]. In the large intestine lactulose is broken down by the action of colonic bacteria primarily to lactic acid, and also to small amounts of formic and acetic acids [56,57]. This acidification favors the formation of the non-absorbable ammonium ion from ammonia and reduces its concentration in plasma. It is unclear in which measure dietary nitrate contributes to the ammonia concentration in the gut and blood, however we suggest the alternative hypothesis that the increased acidification of the colonic content due to the presence of lactulose favors the microbiota conversion of nitrite to NO instead of ammonia by the known acid-dependent mechanism.

### Conclusions

For over 30 years the biological fate of exogenous nitrate could not be accounted for in the excreted nitrogen-containing compounds which amount to approximately 60% of an ingested nitrate dose in humans. Our results support the idea that nitrate is converted to nitrite and then to other reduced nitrogen biomolecules such as NO, ammonia, urea and possibly nitrogen gas by bacteria in the saliva, stomach, small and large intestine. Questions such as how much ammonia is generated from the nitrate-nitrite reduction versus other important processes, such as deamination and the bacterial ureases activity, demands detailed metabolic studies in animals and/or humans. The biological significance of the conversion of dietary nitrates at the intestinal lumen remains to be established. Nevertheless traditional Japanese and Mediterranean diets, which are known to have cardiovascular protective effects, have a mean intake of nitrate per person 2 to 3-fold higher than the typical Western diet (in United States corresponding to about 40–100 mg/day nitrate). Further investigation on the link between symbiotic bacteria, nitrogen oxides metabolism and human health are needed; however it is clear that the biological pathways of nitrogen metabolism in mammals are more complex and more important that envisioned even a few years ago.
