# Correction: Nitrate Reduction to Nitrite, Nitric Oxide and Ammonia by Gut Bacteria under Physiological Conditions

**DOI:** 10.1371/journal.pone.0127490

**Published:** 2015-05-06

**Authors:** Mauro Tiso, Alan N. Schechter

There are errors in the Funding section. The correct funding information is as follows: This study was supported by grants from the intramural program of the National Institute of Diabetes and Digestive and Kidney Diseases. The funders had no role in study design, data collection and analysis, decision to publish, or preparation of the manuscript.
